# Front-Face Fluorescence Spectroscopy and Chemometrics for Quality Control of Cold-Pressed Rapeseed Oil During Storage

**DOI:** 10.3390/foods8120665

**Published:** 2019-12-10

**Authors:** Ewa Sikorska, Krzysztof Wójcicki, Wojciech Kozak, Anna Gliszczyńska-Świgło, Igor Khmelinskii, Tomasz Górecki, Francesco Caponio, Vito M. Paradiso, Carmine Summo, Antonella Pasqualone

**Affiliations:** 1 Institute of Quality Science, Poznań University of Economics and Business, al. Niepodległości 10, 61-875 Poznań, Poland; 2 Department of Chemistry and Pharmacy and Center of Electronics, Optoelectronics and Telecommunications, Faculty of Science and Technology, University of the Algarve, FCT, DQF and CEOT, Campus de Gambelas, 8005-139 Faro, Portugal; 3 Faculty of Mathematics and Computer Science, Adam Mickiewicz University, Uniwersytetu Poznańskiego 4, 61-614 Poznań, Poland; 4 Food Science and Technology Unit, Department of Soil, Plant and Food Sciences, University of Bari, via Amendola 165/a, I-70126 Bari, Italy

**Keywords:** fluorescence, rapeseed oil, multiway analysis, parallel factor analysis (PARAFAC), multivariate regression

## Abstract

The aim of this study was to test the usability of fluorescence spectroscopy to evaluate the stability of cold-pressed rapeseed oil during storage. Freshly-pressed rapeseed oil was stored in colorless and green glass bottles exposed to light, and in darkness for a period of 6 months. The quality deterioration of oils was evaluated on the basis of several chemical parameters (peroxide value, acid value, K_232_ and K_270_, polar compounds, tocopherols, carotenoids, pheophytins, oxygen concentration) and fluorescence. Parallel factor analysis (PARAFAC) of oil excitation-emission matrices revealed the presence of four fluorophores that showed different evolution throughout the storage period. The fluorescence study provided direct information about tocopherol and pheophytin degradation and revealed formation of a new fluorescent product. Principal component analysis (PCA) performed on analytical and fluorescence data showed that oxidation was more advanced in samples exposed to light due to the photo-induced processes; only a very minor effect of the bottle color was observed. Multiple linear regression (MLR) and partial least squares regression (PLSR) on the PARAFAC scores revealed a quantitative relationship between fluorescence and some of the chemical parameters.

## 1. Introduction

The popularity of cold-pressed vegetable oils has increased in recent years due to the tendency of consumers to choose less-processed, healthy foods [1]. The most popular is olive oil with its well established health-promoting properties. Rapeseed oil is a valuable alternative to olive oil. It has a favorable composition of unsaturated fatty acids, from the nutritional point of view; the relation between linoleic (ω-6) and α-linolenic (ω-3) acids in this oil is 2:1. Moreover, it is a rich source of tocopherols and phytosterols [2,3,4].

The quality of oil is affected by oxidation processes that may lead to the loss of nutritional value, deterioration of sensory properties, and formation of toxic products. Oil oxidation proceeds via auto- or photo-oxidation processes, which involve, respectively, triplet or singlet oxygen. Autooxidation of oils involves radical forms of acylglycerols. In photosensitized oxidation, chlorophyll acts as a photosensitizer for the formation of singlet oxygen ^1^O_2_, which reacts directly with double bonds of fatty acids. The hydroperoxides formed during oxidation decompose to produce off-flavor compounds [5]. The rate of oxidation depends on a variety of factors, including chemical composition of oil, temperature, exposure to light, and presence of oxygen.

Rapeseed oil has lower oxidative stability as compared to olive oil [6,7]. Several studies have been performed to assess quality degradation of rapeseed oil during photo- and autooxidation and storage [8,9,10,11,12,13,14]. It was found that the overall quality during storage was influenced by the type of container material (plastics, glass), storage conditions (light, temperature, oxygen availability), and time [13].

Glass is the most popular material used for bottling oils. It is one of the most inert and easy to clean materials. However, colorless glass transmits radiation in the entire visible range, leading to photooxidation of oils and reduction of their shelf-life. Colored glass bottles are used to prevent or limit photooxidation. Green glass bottles are often used to protect oil from light in the 300–500 nm wavelength range [15].

A variety of methods have been used to study oil oxidation and deterioration during shelf-life [15,16,17]. In addition to traditional techniques, spectroscopy coupled with chemometrics was found useful in assessing various aspects of oil quality. Among the spectroscopic techniques, fluorescence provides two main advantages—sensitivity and selectivity, enabling evaluation of minor oil components. Several studies have reported successful applications of oil autofluorescence in the analysis of oil oxidation [18,19,20,21,22,23,24,25,26,27]. Various measurement techniques were used in these studies, including measurements of excitation spectra [18], emission spectra [22,25,26], synchronous fluorescence spectra [19,20,21], and excitation-emission matrices [23,24,27]. This last technique provides the most comprehensive characteristics of fluorescent systems, because excitation-emission matrices are determined by both absorption and emission properties of a sample. Most of the fluorescence studies of oils were conducted using conventional spectrofluorimeters. Recently, a fluorometer with Light Emitting Diode (LED) flashlight excitation and a smartphone was developed for edible oil authentication using a hue-based fluorescence method [28]. 

The aim of the present study was to evaluate quality changes occurring in cold-pressed rapeseed oil during storage for 6 months under different conditions by means of chemical parameters and fluorescence spectroscopy. Parallel factor analysis (PARAFAC) was applied to decompose the excitation-emission matrices. Principal component analysis (PCA) allowed visualization of the relations between differently stored oil samples and the variables describing their properties. The relations between chemical changes and oil fluorescence were studied quantitatively by means of multiple linear regression (MLR) and partial least squares regression (PLSR) performed on PARAFAC scores.

## 2. Materials and Methods

### 2.1. Samples and Storage Conditions

**Figure 1:**
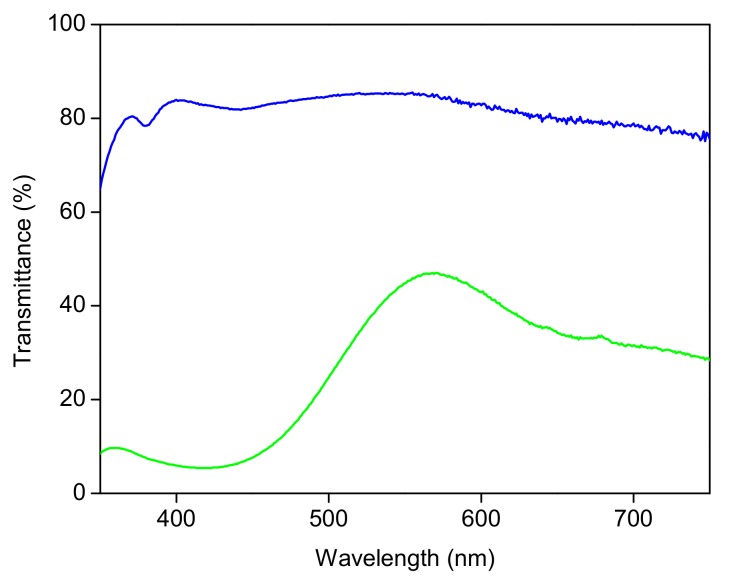
The transmission spectra of colorless (blue line) and green (green line) glass bottles.

Rapeseed oil freshly pressed in a local oil mill was used in the study. A volume of 200 cm^3^ of oil was placed into 250 cm^3^ glass bottles, either colorless or green. The transmission spectra of colorless and green bottles are presented in Figure 1.

The bottling procedure was the same as used in the oil mills. The samples were stored for a period of 6 months (from July to December). The storage conditions were similar to those used in the distribution and marketing of oils. Oil samples were divided into three groups and kept in appropriate conditions: (1) oils in colorless glass bottles, stored without light, marked RD; (2) oils in colorless glass bottles exposed to light, marked RL; (3) oils in green glass bottles, exposed to light, marked RG. The samples were exposed to diffused daylight and additionally for 12 h per day to artificial fluorescent cool white, 4000 K light (Osram light bulb) and illuminance of about 500 lx. Storage under diffused light simulated the conditions of a supermarket shelf. Storage in the dark simulated the warehouse storage. All bottles were stored at ambient room temperatures (18–25 °C). The oils samples were analyzed at the time of packaging and periodically during the 6-month period. Each month, one bottle corresponding to each of the storage conditions was opened and analyzed. 

A total number of 18 samples was used for analysis; a single sample stored for 4 months in a colorless bottle exposed to light was excluded from analysis due to a leaking bottle cap.

### 2.2. Determination of Chemical Parameters

Oxygen concentrations in the bottle headspace and in the oil were determined using a commercially available OxySense 325i system. This instrument enabled non-invasive measurement of the oxygen content through the bottle wall, based on the effect of oxygen on the fluorescence lifetime of optically-excited Tris (4,7-diphenyl-1,10-phenanthroline) ruthenium chloride complex immobilized in a highly stable polymer in the form of an OxyDot oxygen indicator. The system consists of an oxygen concentration analyzer and an EasAlign pen with a built-in temperature sensor and OxyDot indicator. The OxyDot indicators were placed on the internal bottle wall in the headspace and in the oil phase using a transparent adhesive. The calibration of the sensors was performed using pure nitrogen (5.0 purity) and ambient air as standards.

Peroxide values expressed in milliequivalents of active oxygen/kg (meq O_2_/kg of oil) were determined by the iodometric method, according to International Organization for Standardization (ISO) 3960:2007 standard [29].

Acid values were determined according to the standard ISO method 660:2009 [30].

The conjugated dienes and trienes were measured for samples diluted in n-hexane, using a Genesys UV-VIS spectrophotometer (Milton Roy, Houston, USA) and expressed as specific absorption coefficients at 232 (K_232_) and 270 nm (K_270_), according to the standard ISO method 3656:2011 [31].

Polar compounds (PCs) were separated from the oil samples by silica gel column chromatography, according to the Association of Official Analytical Chemists International (AOAC) method no. 982.27 [32]. After elution of the non-polar components with 150 mL of petroleum ether-diethyl ether (87:13, *v*/*v*), the PCs were recovered with 150 mL of diethyl ether. After removing the diethyl ether, the PCs were recovered in tetrahydrofuran (THF) and subjected to high performance size exclusion chromatography (HPSEC) analysis to determine the single classes of substances constituting them. The chromatographic system consisted of a series 200 Perkin–Elmer (Beaconsfield, UK) pump, a 50 µL injector loop (Perkin–Elmer, Beaconsfield, UK), a PL-gel guard column (Polymer Laboratories, Church Stretton, UK) of 5 cm length × 7.5 mm inner diameter, and a series of two PL-gel columns (Polymer Laboratories, Church Stretton, UK) of 30 cm length × 7.5 mm inner diameter each. The columns were packed with highly cross-linked styrene divinylbenzene copolymers with a particle diameter of 5 µm and pore diameters of 500 Å. The detector was a differential refractometer (series 200A, Perkin–Elmer, Beaconsfield, UK). The elution solvent used was THF for high performance liquid chromatography (HPLC) at a flow rate of 1.0 mL/min. The identification and quantification of individual peaks was carried out, as described in previous paper [33]. Three replicates were analyzed per sample.

Total tocopherol content was determined by using a HPLC method. The details of the method are described in Reference [34]. The analysis was carried out using a Waters 600 high-performance liquid chromatograph. Chromatographic separation was achieved at room temperature, using a Symmetry C18 column (150 mm × 3.9 mm, 5 µm), fitted with a µBondapak C18 cartridge guard column (all from Waters, Milford, MA, USA). A mobile phase composed of 50% acetonitrile and 50% methanol was used with a flow rate of 1 mL/min. Samples of oil were weighed (0.0800 g) and dissolved in 1 mL of 2-propanol. Vortex-mixed samples were directly injected into the HPLC column without any additional sample treatment. The injection volume was 20 µL. The eluate was detected using a Waters 474 scanning fluorescence detector, set for emission at 325 and excitation at 295 nm. The emission slit width was 10 nm, fluorometer gain 100, and attenuation 1. Tocopherols were identified by comparing their retention times with those of the corresponding standards. Additionally, a Waters 996 photodiode array detector was used to identify the compounds by their absorption spectra.

Carotenoids were determined using the spectrophotometric method. The concentration of total carotenoids was determined by measuring the absorption of oil dissolved in n-hexane at 450 nm. The molar absorption coefficient at 450 nm for carotenoids: ε = 138730 [L/cm × mol] was used for the calculation of carotenoid concentration [35]. Genesys 2 UV-VIS spectrophotometer (Milton Roy, Houston, USA) was used in these measurements.

Pheophytins content in fresh samples (expressed as mg of pheophytin as per kg of oil) was determined according to official methods and recommended practices of the American Oil Chemists’ Society [36]. The absorbance of the oil sample was read at 630, 670, and 710 nm. In samples exposed to light, low concentration of pheophytins prevented their determination by means of the absorption method. Therefore, relative pheophytins content in all of the samples was determined based on the fluorescence intensity measured at λ_exc_ = 670 nm and λ_em_ = 680 nm, performed using a Fluorolog 3-11 spectrofluorometer (Spex–Jobin Yvon, Palaiseau, France). The relative pheophytins content in all samples studied was expressed as a percentage for further analysis.

### 2.3. Fluorescence Measurements

The fluorescence spectra were obtained using a Fluorolog 3-11 spectrofluorometer (Spex–Jobin Yvon). The excitation-emission matrices (EEMs) were collected by measuring the emission spectra in the 260–700 nm range with the excitation in the 250–500 nm range, with a 10 nm interval. The excitation and emission slit widths were 3 nm. The acquisition step and the integration time were maintained at 1 nm and 0.1 s, respectively. A reference photodiode detector was used to compensate for the source intensity fluctuations. The individual spectra were corrected for the wavelength-dependent response of the system. Front-face geometry was used for undiluted samples in a 10 mm fused-silica cuvette.

### 2.4. Data Analysis

The analysis of covariance (ANCOVA) was used to compare the chemical data for the samples stored in different conditions. Pearson correlation coefficients were calculated to test the correlations between the individual analytical parameters. 

A parallel factor analysis (PARAFAC) was used to decompose excitation-emission matrices into contributions from individual fluorescent components. In PARAFAC, each component consists of one score vector and two loading vectors. The loading matrices contain the spectral excitation and emission profiles of fluorescent components, and the score matrix contains information about the relative contribution of each component to every sample EEMs included into the model [37].

A three-way array of data with the dimensions of 18 × 431 × 26 (number of samples × number of emission wavelengths × number of excitation wavelengths) was used for the PARAFAC. Rayleigh signals in EEMs were removed by replacing them with missing values. Non-negativity constraints on all three modes were applied. The optimal number of components in PARAFAC models was determined based on the core consistency diagnostic (CORCONDIA) and analysis of explained variance [37].

Principal component analysis (PCA) was performed on the X matrix, which contains chemical parameters and fluorescence score data obtained from the PARAFAC. The X data were scaled prior to analysis.

Multiple linear regression (MLR) and partial least squares regression (PLSR) were used to model the relation between the score data obtained from PARAFAC (X) and chemical data (Y). Full cross-validation was applied to all of the regression models. The regression models were evaluated using the adjusted *R*^2^ and the root mean-square error of cross-validation (RMSECV) parameter.

The data analysis was carried out using Solo v. 5.0.1 (Eigenvector Research Inc., Manson, WA, USA) and Unscrambler 9.0 (CAMO, Oslo, Norway) software.

## 3. Results and Discussion

### 3.1. Evolution of Chemical Parameters of Cold-Pressed Rapeseed Oil during Storage

Freshly-pressed rapeseed oil samples were stored in the darkness and exposed to light in colorless and green glass bottles. As shown in Figure 1, colorless glass transmits radiation throughout the visible spectrum in the characteristic absorption range for both carotenoids and pheophytins. Green glass transmission is dependent on the spectral range and it is very low, below 500 nm, and higher in the long-wavelength range where long-wavelength absorption of pheophytins occurs.

**Figure 2:**
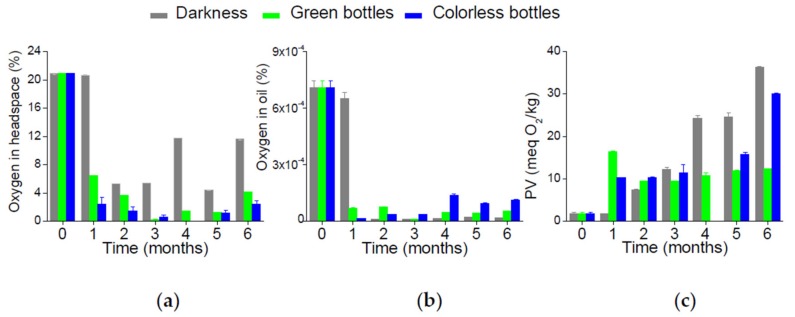

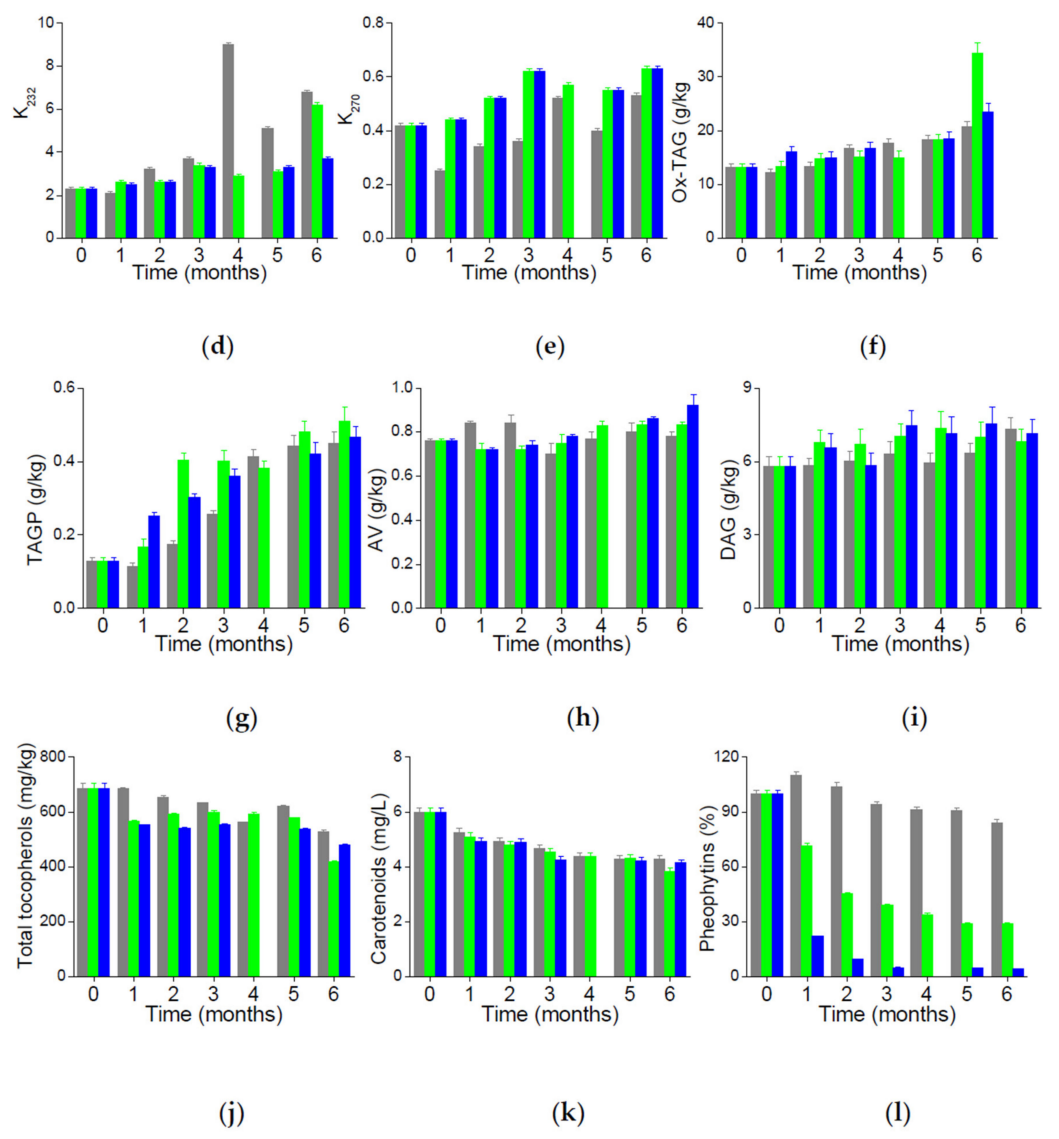
The changes of chemical parameters in the cold-pressed rapeseed oil during the 6 months of storage: (**a**) Oxygen concentration in the bottle headspace; (**b**) oxygen concentration in the oil; (**c**) peroxide value (PV); (**d**) specific absorption at 232 nm, K_232_; (**e**) specific absorption at 270 nm, K_270_; (**f**) content of the oxidized triacylglycerols (Ox-TAG); (**g**) content of polymerized triacylglycerols (TAGPs); (**h**) acid value (AV); (**i**) content of diacylglycerols (DAG); (**j**) total tocopherols; (**k**) carotenoids; (**l**) pheophytins.

The changes in the chemical parameters of the cold-pressed rapeseed oil stored for 6 months in the respective conditions are presented in Figure 2.

Table 1 reports the *p*-values of the analysis of covariance (ANCOVA) of the chemical data obtained for the oil samples stored for 6 months. The effect of time and storage conditions, as well as the results of the first-order interactions on chemical parameters, are presented. The results indicate that the storage time and conditions affected most of the studied oil quality parameters. The statistically significant effects of the storage conditions (*p* < 0.05) were observed for the time evolution of the following parameters: headspace oxygen, peroxide value (PV), K_270_, polymerized triacylglycerols (TAGPs), diacylglycerols (DAG), tocopherols, and pheophytins.

The oxygen concentration in the bottle headspace and in the oil phase was measured in a non-invasive way using a fluorescence sensor. The headspace oxygen content was 20.9% in the freshly-bottled oil samples. A much lower concentration of 0.0007% was detected in the oil phase. The headspace oxygen content decreased during storage at rates depending on the storage conditions (*p* = 0.014), Figure 2a,b. The samples stored in colorless bottles exposed to light had the fastest drop in the headspace oxygen. The oil stored in green bottles revealed a similar decrease in the headspace oxygen concentration. The minimum headspace oxygen concentration, very close to 0.5%, was recorded in these samples when exposed to light in the third month of storage, with some increase observed at longer storage times. The smallest decrease in the headspace oxygen content was observed in oil stored in darkness; the lowest concentration of oxygen, at about 5%, was detected in these samples from the second to the fifth month of the experiment. Similar to the samples exposed to light, higher concentrations of the headspace oxygen were recorded at longer storage times. This minor increase in the oxygen concentrations in the final period of the experiment may result from the oxygen diffusion into the bottle or liberation of oxygen in the radical recombination reactions at the termination step of the oxidation process.

Very low concentrations of oxygen dissolved in oil were recorded for the samples stored in darkness, starting from the second month of the experiment, as shown in Figure 2b.

The peroxide value (PV) evaluates the presence of the primary oxidation products of lipids (hydroperoxides). For a sample of fresh cold-pressed rapeseed oil, the PV was 1.8 meq O_2_/kg and satisfied the requirements of the respective standards for cold-pressed oils [38]. The PV value changes during storage were significantly affected by the storage conditions (*p* = 0.034), as shown in Figure 2c. During the first month, the increase of the PV was much faster in the samples exposed to light (both in colorless and green bottles), as compared to the samples stored in darkness. The peroxide formation rates slowed down in the samples exposed to light after the first month. However, the PV increased almost linearly to the end of the experiment in the samples protected from light. The PV of 15 meq O_2_/kg, allowed by the Codex Standard [38] for virgin oils, was exceeded in the samples stored in darkness after the third month.

The specific absorption coefficient at 232 nm (K_232_) measures the concentration of conjugated dienes, another group of primary oxidation products. The K_232_ value was lower in samples stored in darkness only after the first month (*p* = 0.089), as shown in Figure 2d. From the second month on, the concentration of the conjugated dienes was higher in the samples stored in darkness as compared to the respective samples exposed to light. However, there was no statistically significant effect of the storage conditions on the evolution of this parameter. The lipid hydroperoxides formed during autooxidation are conjugated dienes, whereas photooxidation leads to the formation of both conjugated and nonconjugated dienes [5].

The specific absorption coefficient at 270 nm (K_270_), quantifying the conjugated trienes and the secondary oxidation products (carbonyl compounds), was significantly affected by the storage conditions (*p* = 0.03), as shown in Figure 2e. Its increase was more pronounced and similar in the samples stored in light, independently to the bottle glass color, as compared to those stored in darkness. These observations are in agreement with our previous study; we observed higher increases of K_270_ for the olive oil samples stored under light as compared to those protected from light [39].

The observed changes in the PV, K_232_, and K_270_ parameters indicate that the oxidation was more advanced in the samples stored under light, thus the primary oxidation may have evolved into the secondary. These findings were further verified by the analysis of the evolution of the polar compounds. The substances that are typical oxidation (oxidized triacylglycerols (Ox-TAG), TAGP) and hydrolysis (DAG) products were quantified using the HPSEC method. The analysis of these compounds has already been successfully used for the estimation of the real extent of the oxidative and hydrolytic degradation of various edible oils and fats [40,41].

The evolution of the oxidation products (Ox-TAG and TAGP) is presented in Figure 2f,g. The oxidation product concentration was growing during the storage in all of the storage conditions. The formation of Ox-TAG, Figure 2f, (*p* = 0.609) was not significantly affected by the storage conditions. The Ox-TAG denomination comprises all of the oxidative products deriving from triacylglycerols, which can undergo further polymerization and degradation reactions. It was proposed that these substances could indicate the level of primary oxidation of oils [42].

TAGPs were initially formed at higher rates in the samples exposed to light (*p* = 0.039); however, after 4 months, similar levels of these compounds were observed in all samples, as shown in Figure 2g. The storage conditions significantly affected the evolution of these compounds. TAGPs were proposed as an index of the secondary oxidative degradation, because of their high stability and low volatility [42].

Hydrolytic degradation of oil during storage was evaluated on the basis of the acid value (AV) and DAG content. The AV increased in the samples stored under light throughout the entire duration of the experiment (*p* = 0.543), as shown in Figure 2h. Some fluctuations of the AV were recorded in darkness, although similar values were measured at the beginning and at the end of the storage period. The formation of DAG was affected by the storage conditions (*p* = 0.040), as shown in Figure 2i. Initially, it was higher in the samples exposed to light, while similar levels were recorded in all of the samples at the end of the experiment. 

The progress of oil oxidation also depends on the presence of minor substances. Thus, the concentrations of tocopherols, carotenoids, and pheophytins were measured both in the fresh oil and during storage. Fresh oil was characterized by a relatively high content of vitamin E (total tocopherols) of 684 mg/kg. The tocopherol content decreased with the rates, depending on the storage conditions (*p* = 0.022), as shown in Figure 2j. The fastest decay was noted in the oil stored in colorless bottles. The degradation of tocopherols was the slowest in the samples stored in darkness. The lowest concentration of tocopherols after 6 months was recorded in the green glass bottle. 

In fresh oil the content of carotenoid pigments was 6.0 mg/L. In contrast to tocopherols, the effect of the storage conditions on the decay of carotenoids during the experiment was insignificant (*p* = 0.160), as shown in Figure 2k. 

The evolution of the pheophytins during the storage is of great importance due to their role of a photosensitizer in the photooxidation. In fresh oil, the content of pheophytin pigments of 1.7 mg/kg (pheophytin a) was rather low. The decay of pheophytins was markedly affected by the storage conditions (*p* = 0.001), as shown in Figure 2l. The effect of the bottle glass color was also clearly noticeable. The decay of pheophytins was the most advanced in the samples stored under light in the colorless bottles, as these pigments were partially protected from the photodegradation in the green glass bottles.

Based on the present results, we conclude that the oxidation of rapeseed oil is affected by light exposure mainly during the first few months of storage. Although the studied oil contained only minor amounts of pheophytins, even these speeded up its photooxidation. The green color of bottle glass slowed down the degradation of pheophytins and tocopherols at the initial period of the experiment, having a less pronounced effect on the oxidation during the entire period of storage. Moreover, it seems that oxygen headspace concentration was an important factor that limited the degree and rates of oxidation. It should be noted that due to the high rate of photooxidation, the study in the initial storage period, e.g., up to several days, should provide better insight into the kinetics of this process in colorless and green bottles.

### 3.2. Evolution of Fluorescence of Rapeseed Oil During Storage

**Figure 3:**
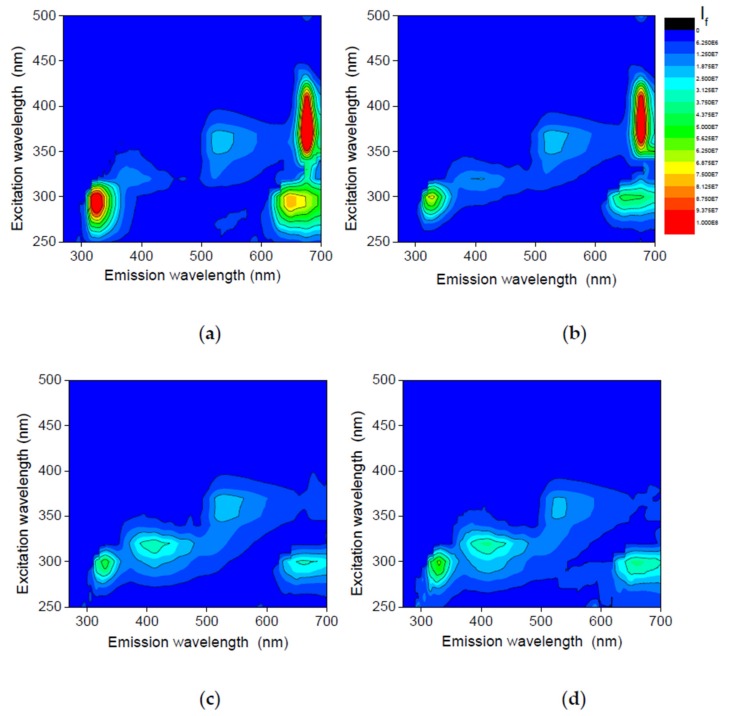
Total fluorescence spectra of freshly-pressed rapeseed oil, R0, (**a**) stored for 6 months in darkness, RD6 (**b**), and exposed to light in green, RG6, (**c**) and colorless, RL6, (**d**) bottles. The same intensity scale.

The fluorescence methods provide two main advantages—high sensitivity and selectivity. Thus, fluorescence enables direct monitoring of minor components of oils, namely tocopherols, pheophytins, and oxidation products. Figure 3 presents the EEMs of fresh rapeseed oil and samples stored for 6 months, exposed to light in colorless and green bottles and stored in darkness. The oils with different oxidation degrees may by discriminated by the intensity of bands ascribed to the particular minor constituents.

Marked differences in the shape and intensity of the fluorescence bands were observed between fresh and stored samples. The most intense fluorescent bands in the short- and long-wavelength regions in the fresh samples correspond respectively to tocopherols (λ_exc_/λ_em_ ca. 300/331 nm) and pheophytins (400/680 nm) [43]. The emission of phenolic compounds may also be observed at the short-wavelength side of this band. The intensity of these fluorescence bands decreased considerably in the stored samples.

The most pronounced differences between the fresh and stored oil spectra were observed in the intermediate spectral zone. Namely, a broad fluorescence band in the intermediate region (λ_exc_/λ_em_ ca. 320/400 nm) appeared during storage. The chemical compounds associated with the emission observed in oils in this range have not been unambiguously identified so far. However, in several studies it was suggested that this emission belongs to oxidation products [22,23,44]. Similarly, we attribute this emission to oxidation products, such as degradation products of pheophytins and/or polar compounds formed in the subsequent oxidation reactions.

The EEMs of all of the oil samples were investigated by means of the PARAFAC method with the objective to resolve the fluorescence landscapes into individual contributions of fluorescent compounds. Based on core consistency and a visual inspection of both the residuals and the loadings, an optimal PARAFAC model was estimated with four components (explained variance 99.1%, core consistency value 70).

**Figure 4:**
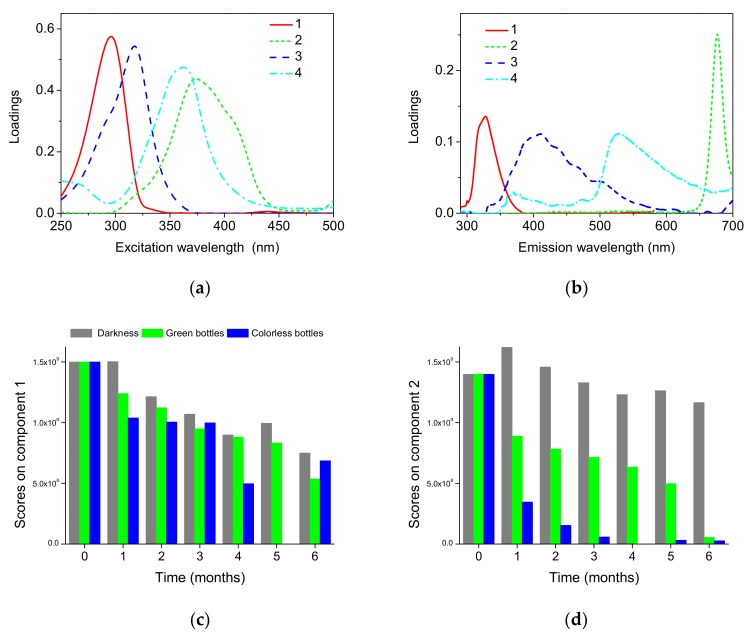

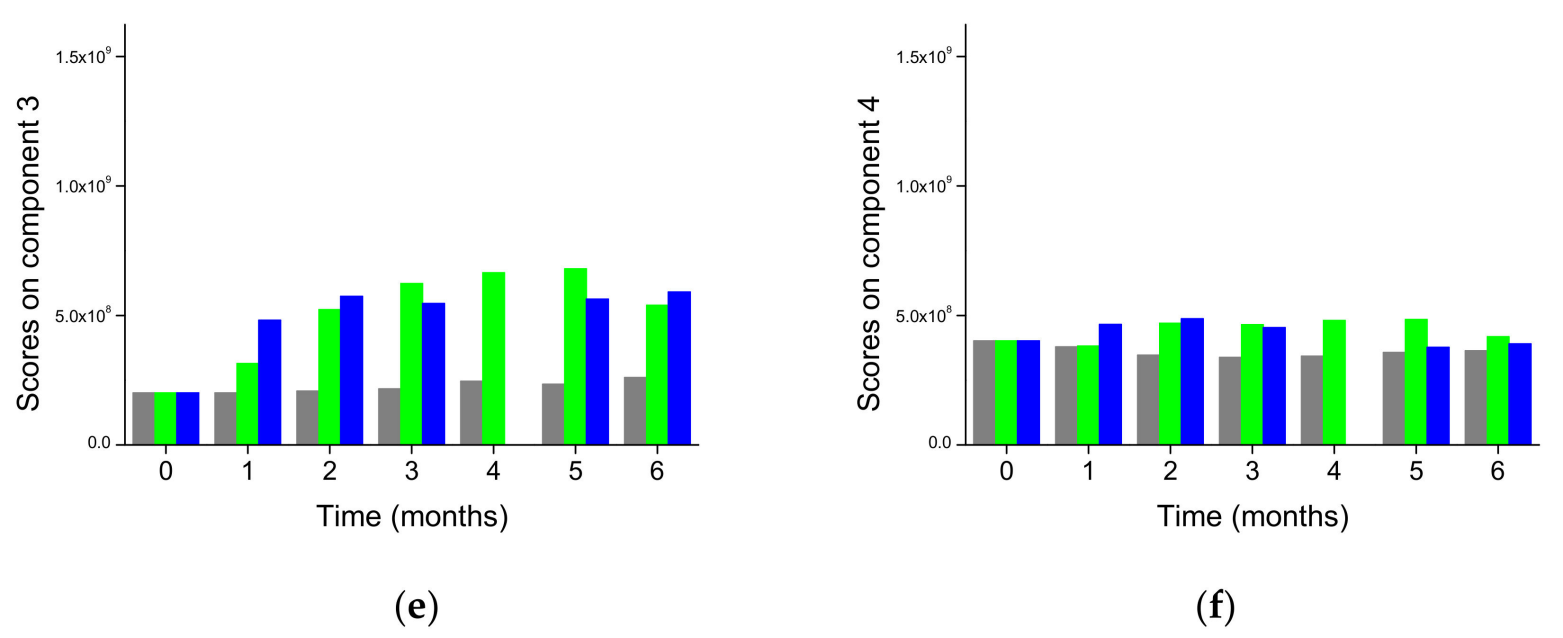
Results of parallel factor analysis (PARAFAC) of total fluorescence spectra of fresh and stored oil samples: Excitation (**a**) and emission (**b**) profiles. Scores vs storage time: scores on component 1 (**c**), scores on component 2 (**d**), scores on component 3 (**e**), scores on component 4 (**f**).

Figure 4 presents the excitation and emission loadings of the four fluorescent components and the respective scores.

The first component appeared at 300/328 nm in excitation/emission and corresponds to tocopherols [43]. The second PARAFAC component appears at 370/677 nm, with a narrow emission band. This component may be ascribed to pheophytins [43]. The third PARAFAC component appears at 320/420 nm in excitation/emission, the fourth at 360/530 nm. The origin of component 3 may be ascribed to compounds formed during oil oxidation. The origin of component 4 was less obvious. The loading of an emission profile of this component, besides the main band, with the maximum at about 530 nm, exhibits additional broad emission with low intensity on the short-wavelength side. This may indicate that emission of some fluorescent components was not fully resolved, and therefore this component corresponds to the more than one chemical compounds.

We showed the contributions of each of the four PARAFAC components in Figure 4c–f. The score values obtained in the PARAFAC decomposition were plotted against the time to explore the progression of fluorescent components throughout the storage. The systematic variations of the score values, corresponding to the respective components, were observed. Thus, the decay of tocopherol (component 1) and pheophytin (component 2) in time was markedly affected by the storage conditions. The effect of bottle color was also noticeable. The decay of component 2 (pheophytin) was the most advanced in samples stored under light in colorless bottles. These pigments were partially protected from the photodegradation in the green bottles. The decay of tocopherol emission was influenced by light and bottle color, particularly in the first months of storage.

The appearance of the oxidation product fluorescence (component 3) was observed for samples stored in light after one month. The contribution of component 4 was affected by storage in a less systematic way. This component was presented in fresh oil and was slightly stronger in samples stored under light and weaker in samples stored in darkness, as compared to fresh oil.

### 3.3. Correlations between Chemical and Fluorescence Data

Next, we used principal component analysis (PCA) to visualize the relationship between differently stored oil samples and the variables describing their properties.

**Figure 5:**
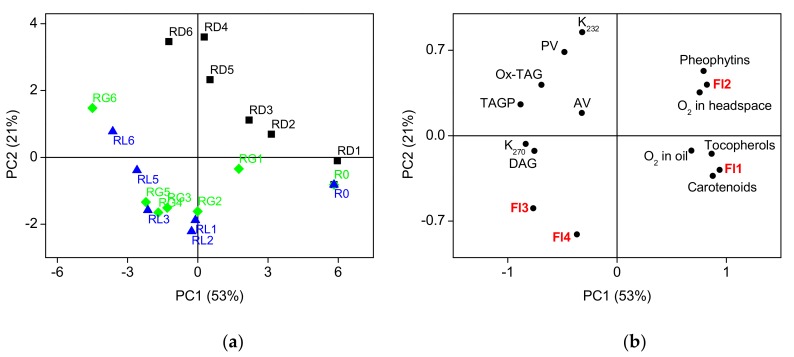
Results of principal component analysis (PCA) of chemical and fluorescent properties of oil samples: scores (**a**), correlation loadings (**b**). Fresh oil—R0; oils in colorless glass bottles, stored without light—RD; oils in colorless glass bottles exposed to light—RL; oils in green glass bottles, exposed to light—RG. Fl1, Fl2, Fl3, Fl4—fluorescent components obtained using PARAFAC. For the abbreviation of chemical parameters see Table 1.

Figure 5 shows the results of the PCA for the analytical parameters and the contributions of the fluorescent components. The distribution of samples of rapeseed oil depended systematically on storage time and conditions, as shown in Figure 5a. Samples exposed to light and protected from light clearly follow different paths during the oxidation. The first two principal components (PC1 and PC2) described 74% of the total data variance.

The first principal component that explained 53% of the total data variance was linked to the time of storage. Samples spread along the PC1 axis, from positive to negative values, according to the storage time. The PC1 was positively correlated (correlation loadings ≥ 0.70) with the contents of tocopherols, carotenoids, and pheophytins, the oxygen concentration in the headspace and oil, and the first (tocopherols) and second (pheophytins) fluorescent components of the PARAFAC decomposition, as shown in Figure 5b. The PC1 was negatively correlated with the K_270_, contents of polar compounds (TAGP, Ox-TAG, and DAG), and PARAFAC components 3. Thus, the progress of oxidation was accompanied by oxygen uptake and the decay of the minor components, tocopherols, carotenoids, and pheophytins, with simultaneous formation of the oxidation products. 

The second principal component (explaining 21% of the total data variance) accounted for both storage time and the variability, due to the storage conditions. The PC2 was positively correlated with the PV and K_232_ and negatively correlated with PARAFAC fluorescent component 4. The oxidative changes in samples of rapeseed oil during storage were clearly affected by storage conditions. Namely, the samples stored in darkness were characterized by higher concentrations of primary oxidation products, as evidenced by the PV, and K_232_.

The significant correlations, evident from PCA analysis and calculated Pearson correlation coefficients (*r*), occurred between the analytical parameters and the contributions of the fluorescent components obtained in PARAFAC. The contribution of the first fluorescent component, identified as tocopherol, was correlated positively with tocopherols, determined by HPLC (*r* = 0.83), carotenoids (*r* = 0.90), pheophytins (*r* = 0.63), and oxygen concentration in oil (*r* = 0.60) and negatively with the formation of Ox-TAG (*r* = −0.77), TAGP (*r* = −0.72), DAG (*r* = −0.63), and the K_232_ (*r* = −0.63) K_270_ (*r* = −0.77). The decrease of the contribution of the second fluorescent component, identified as pheophytin, was correlated positively with the degradation of tocopherols (*r* = 0.72) and pheophytins (*r* = 0.98) and negatively with the increase in K_270_ (*r* = −0.68) and DAG (*r* = −0.62). The contribution of the third fluorescent component, which increased during storage, was positively correlated with K_270_ (*r* = 0.72) and DAG (*r* = −0.64) and negatively with pheophytins (*r* = −0.91) and oxygen in the headspace (*r* = −0.64)). In contrast, the fourth fluorescent component was only negatively correlated with pheophytins (*r* = −0.68).

The decays of the first and second fluorescent components were significantly correlated (*r* = 0.69). The degradation of the first component was correlated with the formation of the third component (*r* = −0.58), while the second component was negatively correlated with the third (*r* = −0.85) and fourth (*r* = −0.59) components. The positive correlation existed also between the third and fourth components (*r* = 0.81).

It should be noted that, among the discussed correlations between fluorescent components and analytical parameters, only those between component 1 and tocopherols and component 2 and pheophytins may be considered as direct. Other correlations were rather indirect and were a consequence of correlations between tocopherols and pheophytins content and respective analytical parameters.

In order to quantitatively model the relationship between the overall fluorescence characteristics (PARAFAC scores) and the chemical parameters, regression analysis was performed using MLR and PLSR methods. We tested the regression models for all of the studied parameters; however, we only present and discuss those models that had acceptable quality (*R*^2^_cal_ > 0.7).

Table 2 presents the results of multivariate calibrations. The best calibration models were obtained for the prediction of pheophytin and total tocopherol content, using both MLR and PLSR on PARAFAC fluorescence scores.

Satisfactory relations were also found between TAGP and fluorescence; however, the prediction error was quite high for this class of substances. The regression analysis did not give satisfactory results for Ox-TAG. A significant relationship was established for K_232_, although these models had the highest relative errors of prediction.

Similarly to the observed correlations between individual fluorescent components and analytical parameters, quantitative relationships are also presented in Table 2, which may be considered as direct only for tocopherols and pheophytins. For other components, such as carotenoids, Ox-TAG, and TAGP, these relationships should be considered as indirect, and may be explained by the previously discussed correlations between various compounds involved in the oxidation processes.

The presented models confirmed that there is a quantitative relationship between fluorescence changes and some of chemical changes occurring during oxidation for the studied samples set. However, due to the indirect nature of these relationships, the respective models cannot be treated as universal models that enable valid determination of analytical parameters in other systems.

## 4. Conclusions

This study was aimed at investigating the potential of fluorescence in combination with chemometric methods for monitoring the rapeseed oil oxidation during storage in different conditions.

The results of chemical analyses revealed that the light exposure affected oxidation of rapeseed oil mainly in the initial period of storage. The minor amounts of pheophytins in oil studied speeded up its photooxidation. The green color of the glass bottle slowed down the degradation of pheophytins and tocopherols during the first few months and gave a less pronounced protective effect on the formation of oxidation products during the whole period of storage.

The PARAFAC of the front-face fluorescence excitation-emission matrices of rapeseed oil uniquely separated four fluorescent components, which had different evolution dynamics throughout the storage period, and revealed oxidative changes occurring in oil during storage. The fluorescence data were quantitatively related to some conventional chemical parameters describing the oxidation status of oil.

The present results show that fluorescence excitation-emission matrices associated with PARAFAC decomposition could be used for the direct monitoring of the oxidative degradation of rapeseed oils during storage.

## Figures and Tables

**Table 1:** Results (*p*-values) of the analysis of covariance (ANCOVA) for the chemical parameters evaluated during the shelf-life of the rapeseed oil.

Parameter	Time	Storage Conditions	Time*Storage Conditions
Oxygen in the bottle headspace	0.378	0.014	0.707
Oxygen in the oil	0.210	0.616	0.081
PV	<0.001	0.034	<0.001
K_232_	0.009	0.089	0.292
K_270_	0.002	0.003	0.333
Ox-TAG	0.002	0.609	0.281
TAGP	<0.001	0.039	0.181
AV	0.003	0.543	0.005
DAG	0.021	0.040	0.381
Tocopherols	0.006	0.022	0.577
Carotenoids	<0.001	0.160	0.488
Pheophytins	<0.001	<0.001	0.105

Peroxide value (PV); acid value (AV); K_232_, K_270_—absorption coefficients at 232 and 270 nm, respectively; oxidised triacylglycerols (Ox-TAG), polymerized triacylglycerols (TAGPs), diacylglycerols (DAG).

**Table 2:** Results of multiple linear regression (MLR) and partial least squares regression (PLSR) of chemical parameters and fluorescent components extracted using PARAFAC.

Parameter ^1^	Regression Method	LV	*R* ^2^_cal_	*R* ^2^_cv_	RMSECV	RE (%)
K_232_	MLR		0.774	0.625	1.1	29
	PLSR	3	0.747	0.640	1.1	29
Ox-TAG (g/kg)	MLR		0.732	0.393	3.9	22
	PLSR	2	0.678	0.498	3.7	21
TAGP (g/kg)	MLR		0.853	0.754	0.061	18
	PLSR	3	0.853	0.799	0.059	17
Tocopherols (mg/kg)	MLR		0.903	0.825	27.2	4.7
	PLSR	3	0.885	0.807	30.2	5.2
Carotenoids (mg/L)	MLR		0.861	0.735	0.25	5.4
	PLSR	2	0.836	0.765	0.25	5.4
Pheophytins (%)	MLR		0.984	0.965	7.03	13.7
	PLSR	3	0.984	0.972	6.65	12.4

^1^ For the abbreviation of chemical parameters see Table 1. Latent variables (LVs) of PLSR models; *R*^2^cal—determination coefficient of calibration; *R*^2^cv—determination coefficient of validation; root mean-square error of cross-validation (RMSECV);— relative error (RE).
